# Analysis of Post-Deposition Recrystallization Processing via Indium Bromide of Cu(In,Ga)Se_2_ Thin Films

**DOI:** 10.3390/ma14133596

**Published:** 2021-06-28

**Authors:** Deewakar Poudel, Benjamin Belfore, Tasnuva Ashrafee, Shankar Karki, Grace Rajan, Angus Rockett, Sylvain Marsillac

**Affiliations:** 1 Virginia Institute of Photovoltaics, Old Dominion University, Norfolk, VA 23529, USA; dpoud001@odu.edu (D.P.); bbelf001@odu.edu (B.B.); tashr001@odu.edu (T.A.); skark002@odu.edu (S.K.); gcher002@odu.edu (G.R.); 2 Department of Metallurgical and Materials Engineering, Colorado School of Mines, Golden, CO 80401, USA; arockett@mines.edu

**Keywords:** copper indium gallium selenide, post-deposition treatment, recrystallization, indium bromide

## Abstract

Cu(In,Ga)Se_2_ (CIGS) thin films were deposited at low temperature (350 °C) and high rate (10 µm/h) by a single stage process. The effect of post-deposition treatments at 400 °C and 500 °C by indium bromide vapor were studied and compared to the effect of a simple annealing under selenium. Structural, electrical, and chemical analyses demonstrate that there is a drastic difference between the different types of annealing, with the ones under indium bromide leading to much larger grains and higher conductivity. These properties are associated with a modification of the elemental profiles, specifically for gallium and sodium.

## 1. Introduction

Copper indium gallium diselenide (CIGS) thin films have exhibited widespread photovoltaic (PV) applications due to their tunable bandgap and high absorption coefficient [1]. The devices can be fabricated with processes leading to very high efficiency, such as the conventional three stage process, or by processes that can be more easily implemented industrially, such as a single stage process, which can fundamentally be performed at higher deposition rate and lower cost [2]. One of the current emphases is to find a process that can combine the advantages of both types of fabrication processes, retaining high efficiency device at low cost in order to contend with other commercially available PV technologies. Powerful post-deposition treatments have already been applied to other PV technologies, such as cadmium telluride (CdTe), by heating CdTe in the presence of CdCl_2_, resulting in the recrystallization of CdTe [3,4,5]. It would be of course of interest to develop such a process for CIGS, allowing post-deposition treatment to recrystallize the thin films with the goal of enhancing the semiconductor quality [6].

In this study, to investigate the feasibility and conditions required for post-deposition recrystallization, CIGS films deposited at 350 °C were recrystallized at various temperatures under InBr_3_ vapor treatment. The resulting films were analyzed for their composition and morphological transformations.

## 2. Materials and Methods

A molybdenum bilayer was first deposited by DC magnetron sputtering on soda-lime glass (SLG) substrates at constant power density of 7.4 W/cm^2^. The bottom layer was deposited at higher Ar pressure (1.06 Pa or 8 mTorr), while the top layer was deposited at a low Ar pressure (0.53 Pa or 4 mTorr), resulting in a tensile/compressive stress dipole. The resulting combined thickness of Mo was about 800 nm [7]. CIGS thin films were then grown at a substrate temperature of 350 °C using a single stage co-evaporation process. The as-deposited samples were Cu-poor (Cu/III or Cu/(In+Ga) ratio below 1). The substrate temperature and all source temperatures were kept constant. After the deposition, the films were loaded into a small annealing chamber. Within that annealing chamber, a small quartz tube was loaded with the sample and charge to ensure more fluxing agent interacted with the films. A small charge (5 mg) of InBr_3_ as well as elemental selenium (50 mg) was loaded in the small tube with the films (referred to as InBr_3_-annealed). The elemental selenium is necessary to prevent evaporation of selenium from the films. The chamber was then pumped down to less than 1.33 Pa (10 mTorr) and annealed for 30 min at either 400 °C or 500 °C. The annealing temperatures of 400 °C and 500 °C were chosen instead of the frequently used temperatures of 550–600 °C in order to demonstrate that recrystallization and grain growth could be observed by InBr_3_ treatment even at low temperatures. Note that for the 500 °C runs, additional references samples, with just elemental selenium, were fabricated to differentiate the effect of pure annealing from the recrystallization process. The samples were then left to cool down. After cooling, they were rinsed with deionized water to eliminate residual surface phases.

Film compositions were measured by X-ray fluorescence (XRF) (Solar Metrology, System SMX, Holbrook, AZ, USA) with Cu X-ray tubes. Surface and cross-section morphological analysis was performed by scanning electron microscopy (SEM) (JEOL JSM-6060LV, Peabody, MA, USA) (accelerating voltage of 5–30 kV, resolution of 3.5 nm (HV mode), 4.0 nm (LV mode), magnification of 5X-300000X). The crystallographic structure analysis was done by symmetric θ–2θ X-ray diffraction (XRD) (Rigaku Miniflex II Benchtop X-ray Diffractometer, Austin, TX, USA), using Cu Kα radiation source, λ = 1.54 A°). Electrical properties of the films were measured by Hall effect (Ecopia, HMS 3000 Hall Measurement System, Gyunggi-Do, Korea) measurements performed on films deposited on glass. The elemental composition profile of the films was measured by dynamic secondary ion mass spectrometry (SIMS), using an ION-TOF TOF SIMS V instrument (Muenster, Germany). Dual-beam depth profiling was completed with a 100 × 100 μm^2^ imaged area, and a 300 × 300 μm^2^ sputter-beam raster area. A 30 keV Bi_3_^+^ beam was used as the analysis beam, which was scanned over the center of the sputtered crater. A 2 keV Cs^+^ beam with a current of the 75 nA was used to sputter the sample for depth profiling.

## 3. Results and Discussion

### 3.1. Post-Deposition Recrystallization at 400 °C

**Figure 1:**
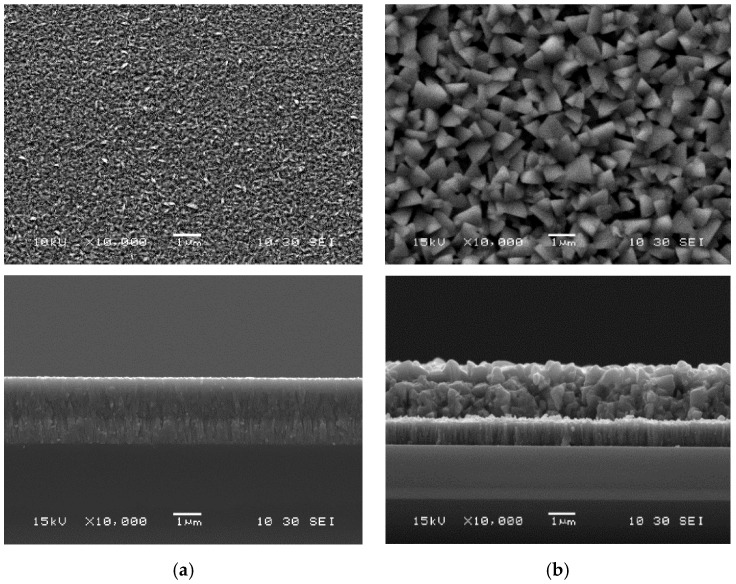
Scanning electron microscopy micrographs (surface and cross section) of CIGS films: as-deposited (**a**) and recrystallized in InBr_3_ at 400 °C (**b**).

The as-deposited samples were annealed at 400 °C in an InBr_3_ environment for a duration of 30 min. The surface and cross-sectional SEM images of CIGS samples before and after recrystallization are shown Figure 1. As one can see, a drastic change in morphology can be observed for the films after recrystallization by InBr_3_, even for these low temperatures of 400 °C. The films evolved from undefined structure for the as-deposited to larger grains. One can also observe an increase in thickness for the films after annealing. 

**Figure 2:**
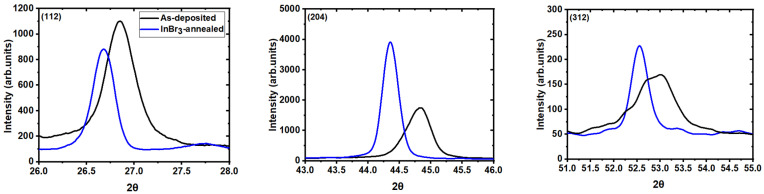
XRD plots of the three key CIGS peaks ((112), (204), and (312)) for the as-deposited (black) and InBr_3_ annealed at 400 °C (blue) CIGS samples.

XRD measurements were completed on the as-deposited and recrystallized films to analyze how the crystalline structure of the films changed with InBr_3_ treatment (Figure 2 and Table 1). The as-deposited films XRD plots had several CIGS characteristics peaks (ICDD Database, Card No-00-035-1102) [8], whose positions corresponded to a composition of Ga/III = 0.24 (where Ga/III is the Ga/(Ga+In) atomic ratio), in good agreement with the XRF measurements. The films were slightly (220)/(204) oriented. After recrystallization, one could observe a change in all parameters. There was notably a decrease in the full width at half maximum (FWHM), from 0.29° to 0.17° for the (112) peak, for example. This correlated well with the increase in grain size observed by SEM.

A change in preferential orientation could also be seen, with the films having a higher degree of preferential orientation along the (220)/(204) direction after recrystallization. All the peak positions also changed to a lower angle, from 26.8° to 26.6° for the (112) peak, for example, corresponding to a decrease in Ga/III ratio [9]. This was correlated with the results from XRF, indicating a reduction of Ga/III ratio from 0.24 to 0.05. The XRF results also indicated that the films became more copper-poor, which means that indium was added to the films during the InBr_3_ vapor treatment. On the other hand, Hall effect measurements revealed an improvement of the conductivity from 1.9 × 10^−3^ ohm^−1^·cm^−1^ for the as deposited films to 35.7 × 10^−3^ ohm^−1^·cm^−1^ for the recrystallized films, due to both an increase in the carrier concentration (N_A_) and mobility (µ) (Table 1).

### 3.2. Post-Deposition Recrystallization at 500 °C

**Figure 3:**
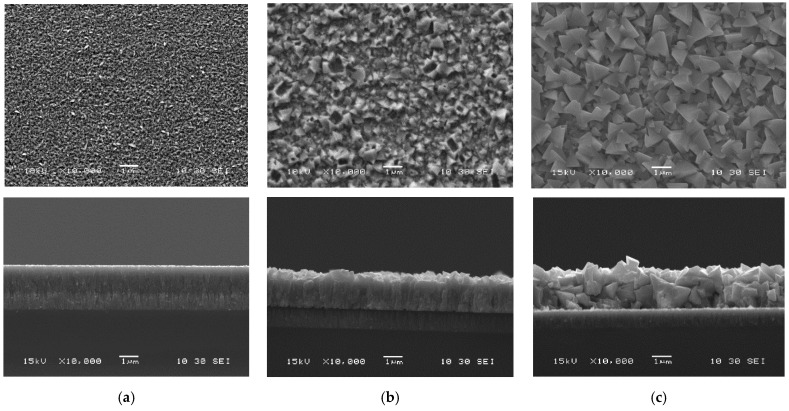
Scanning electron microscopy micrographs of CIGS films: as-deposited (**a**), recrystallized in Se (**b**), and recrystallized in InBr_3_ (**c**) at 500 °C for 30 min.

The surface and cross-sectional SEM images of CIGS samples before and after recrystallization are shown Figure 3. As mentioned, the as-deposited CIGS samples were split into two groups: one recrystallized at 500 °C in Se for 30 min, and one recrystallized at 500 °C in the InBr_3_ environment for 30 min. Cross-section micrographs in combination with top surface micrographs revealed the increase in grain dimension for all annealed films. As compared to the as-deposited, the Se annealed samples illustrated only a slight change in microstructure, mostly seen in the surface SEM. Looking at the SEM micrographs of the films annealed in InBr_3_ atmosphere, one can see a significant microstructural evolution with larger grains. Comparing the Se and InBr_3_ annealed samples, one can see that when only higher temperature was applied, there was indeed a change in the grain size but not much change in the surface, whereas with the InBr_3_ there was enhanced surface faceting but also much higher surface roughness. This seems to indicate that a different process is occurring when InBr_3_ is used, which yields not only grain enhancement but also transport of matter.

**Figure 4:**
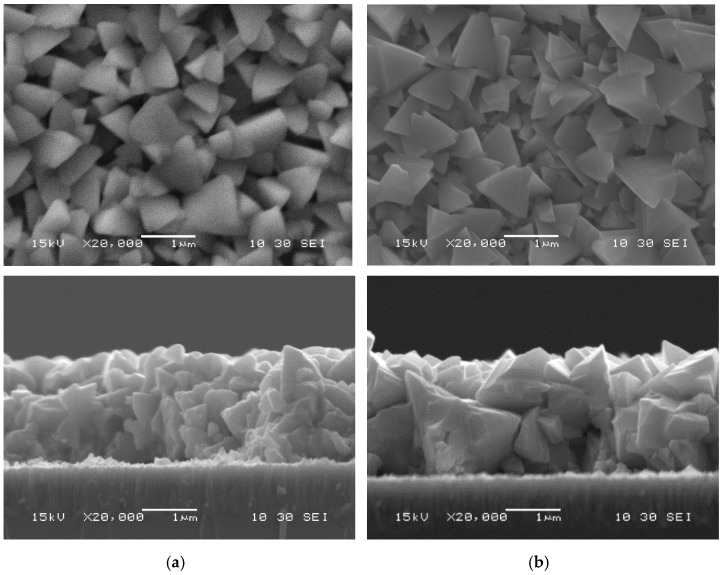
Scanning electron microscopy photographs of CIGS films comparing recrystallization in InBr_3_ for 30 min at (**a**) 400 °C and (**b**) 500 °C.

The difference in grain formation by InBr_3_ vapor treatment for the two different annealing temperature (400 °C and 500 °C) is illustrated Figure 4. While the surfaces look reasonably similar for both temperature, one can see from the cross-sections that much larger grains were obtained at 500 °C, with some grains nearly as large as the full film thickness.

**Figure 5:**
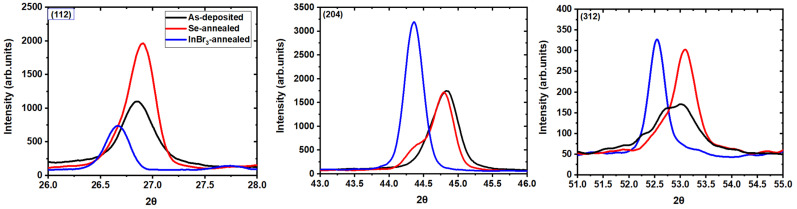
XRD plots of the three key CIGS peaks ((112), (204) and (312)) for the as-deposited (black), Se-annealed (red), e and InBr_3_ annealed (blue) at 500 °C CIGS samples.

XRD measurements were completed on all three films to elucidate the structural evolution within the films and are illustrated in Figure 5 and Table 2. No clear phase separation was observed for the (112) orientation. The noticeable change between the as-deposited and Se-annealed films was the formation of two distinct phases, as seen by the formation of a shoulder in the (220)/(204) XRD peak. Looking at the preferential orientation, one can see that the as-deposited films were preferentially oriented along the (220)/(204) direction, the Se annealed along the (112) direction, and the InBr_3_ annealed along the (220)/(204) direction. The full width at half maximum (FWHM), which is inversely proportional to the grain size as indicated by Scherrer’s formula, showed clearly an increase in grain size from the as-deposited films to the Se annealed films, and from the Se annealed films to the InBr_3_ annealed, in good agreement with SEM micrographs.

To assess the evolution of the elemental composition with the processes, XRD calculation and XRF measurements were performed (Table 2). The results showed the same composition for the as-deposited and the Se-annealed films by XRF, while the XRD shows that there were two peaks, one with lower Ga content and one with higher Ga content, indicating a Ga redistribution in the film. On the other hand, the InBr_3_-annealed samples became indium rich to some extent, as indicated by both XRF and XRD. To assess the evolution of the electrical properties of the films due to various annealing, Hall effect measurements were performed on CIGS thin films deposited directly on SLG along with the other samples and enduring the same thermal treatment. The results indicated an enhancement of the conductivity from 1.9 × 10^−3^ ohm^−1^·cm^−1^ for the as deposited films, through 10.1 × 10^−3^ ohm^−1^·cm^−1^ for the selenium annealed films, to 70.5 × 10^−3^ ohm^−1^·cm^−1^ for the recrystallized films due to both an increase in the carrier concentration (N_A_) and mobility (µ).

**Figure 6:**
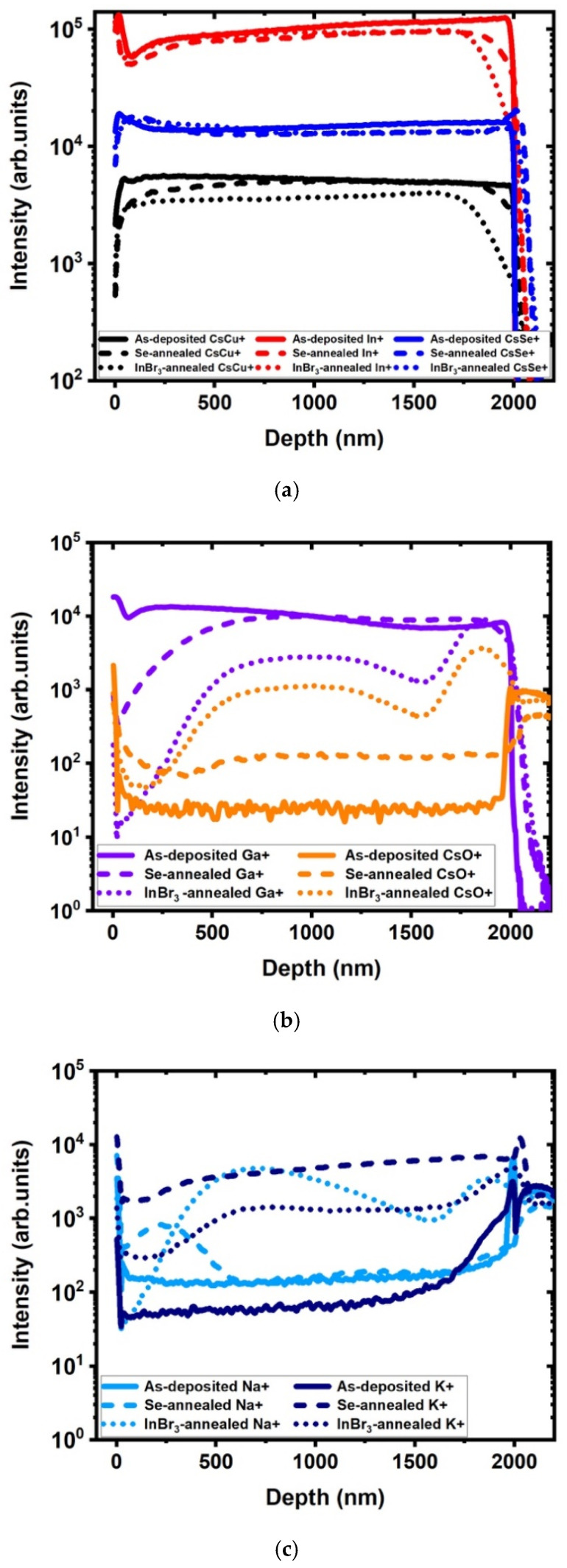
Secondary ions mass spectrometry (SIMS) depth profile (positive ions) for the as-deposited, Se-annealed, and InBr_3_ annealed at 500 °C CIGS samples: (**a**) Cu, In, and Se ions; (**b**) Ga and O ions; (**c**) alkali ions (as-deposited: full line; Se annealed: dashed line; InBr_3_ annealed: dotted line).

The samples were also investigated by dynamic secondary ion mass spectrometry (SIMS) to assess the impact of the recrystallization process on elemental depth profile. The depth profiles for the positive ions of the main element for all the samples are shown in Figure 6. For the as-deposited films, the constant ions profiles for CIGS main elements (Cu, In, Ga, Se) and the presence of alkali (Na^+^ and K^+^) were consistent with CIGS films deposited by a single stage process, since all the temperature sources (and therefore the rates) were kept constant throughout the process.

After Se annealing, one can see that there was no change in three of the main element profiles, namely Cu, In, and Se. One can see, however, a reduction in the Ga concentration at the surface compared to the reference, associated with a small increase in oxygen content, at the surface and in the bulk of the films (Figure 6b). The sodium profile changed much more drastically (Figure 6c), with an increase of the intensity by nearly two orders of magnitude, while the potassium profile only changed closer to the surface, with an increase in intensity. The change in sodium concentration is typically associated with the higher temperature from the recrystallization, as sodium diffuses from the glass, through the molybdenum, into the CIGS layer [10,11]. The change in Ga profile was more unusual, as Ga tends to not inter-diffuse much, even at 500 °C. This property was actually used in devising the classical three-stage process, where no Ga is deposited during the 2nd stage, leading to a lack of Ga in the middle of the device and an enhanced CIGS device efficiency [12,13]. It is therefore likely that this change in Ga profile was related to the change in grain size and reordering of the elemental matrix, as seen by XRD and SEM. It is also possible that selenium acted as a fluxing agent along with Na [14], redistributing slightly the Ga at the surface. Indeed, XRF indicated that the overall composition did not change.

After InBr_3_ annealing, there was still no noticeable change in the profile of Cu, In, and Se. However, a drastic change in all the other elements profiles occurred (Figure 6b,c). There was an overall decrease in the Ga intensity compared to the reference, most importantly at the surface (similarly to the Se anneal) but also throughout the depth of the film. This was accompanied by a similar change in the oxygen profile, indicating that both changes were likely correlated in their mechanism and occurrence. This decrease in gallium signal intensity could be linked to the decrease in gallium concentration observed by both XRF and XRD. Changes in Ga profile in CIGS due to the fabrication process are known and have been observed when changing, for example, from a one-stage to a three-stage process [15], when modifying the copper content in the films [16] or when modifying the process temperature [17]. However, when designing intentionally the Ga profile, as in the three-stage process [12,17], one generally tries to have a Ga-rich layer at the back of the device (to form an electron back-reflector) and at the front of the device (to enhance open circuit voltage) while keeping the central part at a lower Ga content (to enhance current collection) [12,17]. With the current process, however, the films ended up with a low Ga content at the front, which will have to be modified as it is not ideal for device fabrication.

The oxygen atoms come from the chamber, which was only evacuated to the level of 1.33 Pa (10 mTorr); it is interesting to notice though that a much lower level of oxygen was integrated during selenium annealing. This could be due to the important change in grain morphology during recrystallization, exposing more grain surfaces to the environment, but also to the hygroscopic nature of InBr_3_. One can also wonder why the recrystallization process affected mostly the gallium and not the other main elements (Cu, In, and Se). A possible explanation might come from the negative ions’ SIMS profiles (not shown), where a small bromine signal could be observed in the films. Due to the presence of bromine, it is possible to have reaction with elements of the matrix. While both InBr_3_ and SeBr_4_ are volatile species, they were being continually replenished by the added Se and InBr_3_ during the annealing process. On the other hand, CuBr has a fairly low vapor pressure. At 500 °C, the vapor pressure of CuBr is less than 1.33 Pa (10 mTorr). On the other hand, the normal boiling point of GaBr_3_ is 279 °C. The high vapor pressure of GaBr_3_ might cause it to be rapidly volatilized and would explain the observed depletion [18,19]. The Na profile mostly followed the Ga profile in its shape but saw an increase in concentration instead of a decrease, whereas the K profile showed a small decrease in concentration compared to the selenium anneal. Looking at the electrical measurements, one can see that there was an increase in carrier concentration from the as-deposited films to the InBr_3_ recrystallized films, which could be correlated to the increase in Na concentration [20]. A higher Na concentration is actually positive, as Na is associated with enhanced device characteristics. The appropriate ratio of Na and K is actually critical for higher efficiency CIGS device fabrication but can be controlled via post-deposition treatments [11,12,16,17].

## 4. Conclusions

The capacity to perform post-deposition treatments on polycrystalline solar cells to enhance their properties is critical for potential industrial development of these technologies, as demonstrated by CdTe solar cells. In this paper, a process based on a similar concept, but replacing the group CdTe/CdCl_2_ with CIGS/InBr_3_, was studied. Clear modifications of the grain size and of the electrical properties were observed by SEM, XRD, and the Hall effect. These changes would seem to go in a positive direction for the fabrication of a solar cell. However, they are accompanied by major changes in the composition too, especially for the gallium and the sodium content, clearly seen by SIMS when comparing with the reference samples but also with the samples annealed only under selenium. The enhanced sodium content is generally beneficial for CIGS solar cells (notably for the open circuit voltage), but the change in gallium profile, if uncontrolled, could yield very poor device results. Better control of the process will therefore be required, either by changing the temperature or the amount of InBr_3_ introduced, or by pre-emptively depositing a layer with a higher gallium concentration at the front. Without modification of the gallium and sodium profiles, it is unlikely that high efficiency devices can be fabricated.

## Figures and Tables

**Table 1:** XRD data analysis, XRF, and Hall effect measurements for the samples annealed at 400 °C.

Characterizations	Parameters	As-Deposited			InBr_3_-Annealed		
XRD	Peaks	(112)	(220)/(204)	(312)	(112)	(220)/(204)	(312)
	Angle (°)	26.8	44.8	52.9	26.6	44.3	52.5
	Intensity (counts)	1092	1714	170	890	3900	227
	FWHM (°)	0.29	0.39	0.48	0.17	0.19	0.29
	Ga/III	0.24			0.07		
XRF	Ga/III	0.24			0.05		
	Cu/III	0.85			0.72		
Hall effect	Carrier Concentration (N_A_) (10^16^ cm^−3^)	0.5			5.2		
	Mobility (µ) (cm^2^/s)	2.4			4.3		
	Conductivity (10^−3^ ohm^−1^·cm^−1^)	1.9			35.7		

**Table 2:** XRD data analysis, XRF, and Hall effect measurements for the samples annealed at 500 °C.

Characterization	Parameters	As Deposited		Se-Annealed		InBr_3_-Annealed	
XRD	Peaks	(112)	(220)/(204)	(112)	(220)/(204)	(112)	(220)/(204)
	Angle (°)	26.8	44.8	26.8	44.3	26.6	44.3
					44.7		
	Intensity (counts)	1092	1714	1875	512	719	3221
					1688		
	FWHM (°)	0.29	0.39	0.19	0.40	0.19	0.22
					0.22		
	Ga/III	0.24		0.09		0.08	
				0.30			
XRF	Ga/III	0.24		0.24		0.04	
	Cu/III	0.85		0.84		0.70	
Hall Effect	Carrier Concentration (N_A_) (10^16^ cm^−3^)	0.5		1.8		7.1	
	Mobility (µ) (cm^2^/s)	2.4		3.5		6.2	
	Conductivity (10^−3^ ohm^−1^·cm^−1^)	1.9		10.1		70.5	
